# Supplementary material for: Influence of Laser Energy Input and Shielding Gas Flow on Evaporation Fume during Laser Powder Bed Fusion of Zn Metal
Source: Materials (Basel). 2021 May 20;14(10):2677. doi: 10.3390/ma14102677 (PMC8160986; doi:10.3390/ma14102677)
Supplement: Supplementary file 1 [file materials-14-02677-s001.zip › Thermal physical parameters used in simulation.pdf]

Table 1 Thermal physical parameters used in simulation

Parameters	Unit	Zn	TC4
Density	*kg/m³*	740(293.15K); 6553(700K); 6508(750K); 6464(800K); 6420(850K); 6376(900K); 6332(950K)	4440
Specific heat capacity	*j/kg*K*	[22.4+0.0105*T(298-692.7K)]/0.065; 483.1(692.7-1180.15K); 320(>1180.15K)	611(293.15K);713(873K); 771(1473K)
Thermal conductivity	*W/m*K*	113(293.15K);96(683.65K); 61(692.65K);57(1023.15K)	6.8(293K); 13.7(873K) 21.7(1473K)
Viscosity	*kg/m*s*	0.003737(700K); 0.002883(800K) 0.002356(900K) 0.002005(1000K) 0.001756(1100K)	0.005
Molar mass	*kg/mol*	65	411
Heat of fusion	*J/kg*	100900	6340000
Thermal expansion	*1/K*	0.89*10^-6(293-673K)	0.0001
Melting point	*K*	692.65	1928
Boiling point	*K*	1180.15	3375
Surface tension	*mN/m*	782(692.65K);755(723.15K); 751(773.15K)	1.68-(2.6*0.0001)*(T-1878)
Surface tension coefficient	-	0.0004	0.00026
Laser absorptivity	-	36%(Bulk);73%(Powder)	73%(Powder)
Latent heat of vaporation	*kJ/mol*	115.3	12.9
